# Case Report : Li-Fraumeni Syndrome with Central Nervous System Tumors in Two Siblings

**DOI:** 10.1186/s12887-021-03070-8

**Published:** 2021-12-27

**Authors:** Zishi Fang, Yan Su, Hailang Sun, Ming Ge, Zhan Qi, Chanjuan Hao, Suyun Qian, Xiaoli Ma

**Affiliations:** 1 Medical Oncology Department, Pediatric Oncology Center, Beijing Children’s Hospital, National Center for Children’s Health, Beijing Key Laboratory of Pediatric Hematology Ocology, Key Laboratory of Major Diseases in Children, Ministry of Education, Hematology Oncology Center, Capital Medical University, Beijing, 100045 China; 2 Department of Neurosurgery, Beijing Children’s Hospital, National Center for Children’s Health, Capital Medical University, Beijing, China; 3 Department of Medical Genetics, Capital Institute of Pediatrics, Beijing Children’s Hospital, National Center for Children’s Health, Beijing, China; 4 Pediatric Intensive Care Unit, Beijing Children’s Hospital, National Center for Children’s Health, Capital Medical University, Beijing, China

**Keywords:** Li-Fraumeni Syndrome, Pediatric Oncology, TP53 Gene Mutation, Choroid plexus carcinoma, Glioblastoma

## Abstract

**Background:**

Li-Fraumeni syndrome (LFS) is a rare autosomal dominant cancer predisposition syndrome caused by germline TP53 gene mutations. It is characterized by high risk of early-onset cancer, and has been confirmed as associated with multiple tumors clinically. So pediatricians should be more alert to LFS in children with tumors. Choroid plexus carcinoma (CPC) is a rare, malignant tumor which account for less than 1% of all central nervous system (CNS) tumors. However, when such tumorigenesis occurs, it is important to be vigilant for the presence of LFS.

**Case presentation:**

The first patient is a 32-month-old boy admitted for convulsions and then was found intracranial space-occupying lesion. Underwent operation, he was diagnosis as choroid plexus carcinoma (WHO Grade III). After 5 months, his elder sister, a 13-year-old girl, was brought to emergency department for confusion and intermittent convulsions. Surgery was performed immediately after head CT examination found the lesion. The pathology result indicated glioblastoma. Because the siblings of the same family have successively suffered from malignant tumors, we performed genetic testing on this family. TP53 gene mutation occurred in both children of these two cases from their father, and their other brother was not spared either. So the two siblings both met the diagnostic criteria of LFS. Then they all received systematic anti-tumor therapy, and follow-up hitherto.

**Conclusion:**

Here we reported a rare LFS case that two siblings were inherited the same TP53 germline mutations from their father. They suffered from choroid plexus carcinoma and glioblastoma and were finally diagnosed with LFS. In this LFS family, the primary tumors of the two children were both central nervous system tumors, which were not reported in the previous literature. It is suggested that clinicians should be alert to LFS related tumors, which is helpful for early diagnosis. Timely detection of TP53 gene is an important way for early diagnosis of LFS, especially in children with tumor. The incidence of secondary tumor in LFS patients is significantly higher, and other family members of the LFS patient also have an increased risk of suffering from the tumors. Therefore, early diagnosis and timely tumor surveillance can obtain better therapeutic effect and prognosis for both proband and their family.

## Background

Li-Fraumeni syndrome (LFS) is a rare autosomal dominant cancer predisposition syndrome characterized by high risk of early-onset cancer and multiple cancer types [1]. Several kinds of tumors are closely associated with LFS including sarcomas, premenopausal breast cancer, brain tumors and adrenocortical carcinoma. Leukemia, lymphoma and nephroblastoma are also confirmed to be relevant to LFS in some cases [2]. Germline mutations in the tumor suppressor gene TP53 are widely recognized as the pathogenic gene of LFS now [3]. Identification of related tumors and timely genetic testing are the key to the early detection of LFS. It is also a way to find the second tumor and monitor other members of the family. Here we reported a rare LFS case that two siblings were inherited the same TP53 germline mutations from their father. They suffered from choroid plexus carcinoma and glioblastoma and were finally diagnosed with LFS. Unfortunately, the TP53 mutation was also found in their brother. It is seldom reported that all offspring carry the TP53 mutant gene in one family, and even rarer is that the two siblings both have central nervous system tumors and the onset time is quite similar. We aim to emphasize the importance of early diagnosis of LFS through the case and share the management of LFS patients and their family.

## Case presentation

### Case 1

**Fig. 1:**
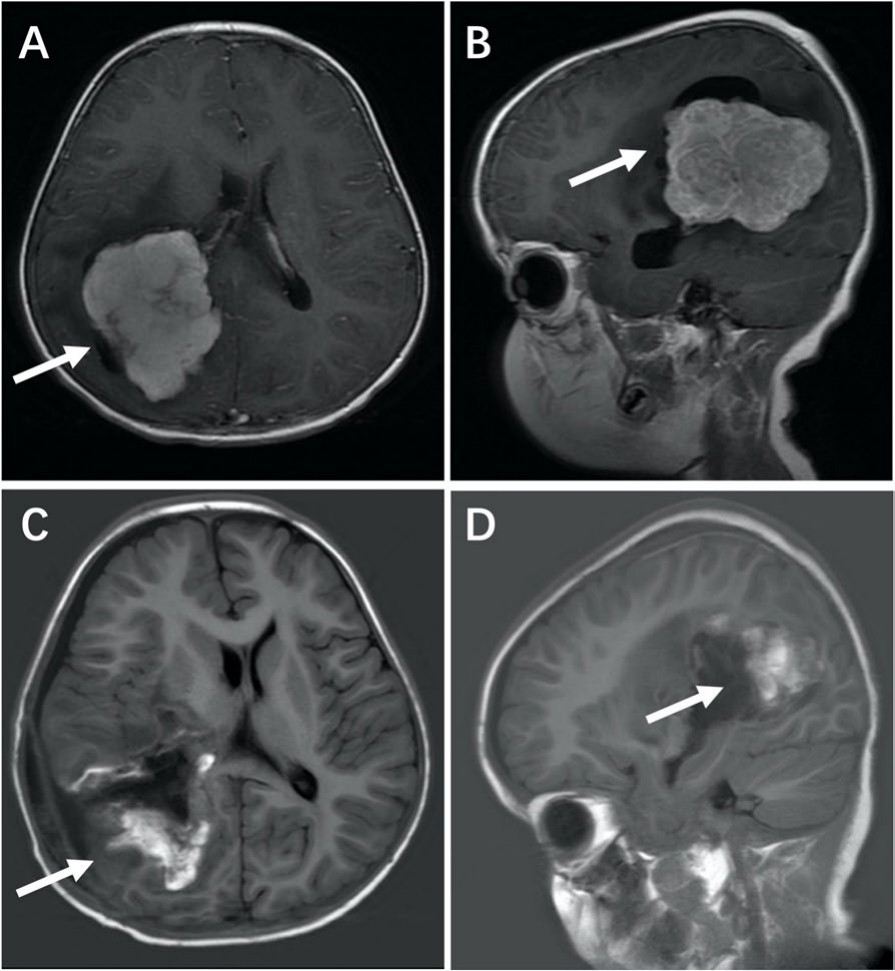
Brain MRI before the operation; **(A, B)** A giant (4.9cm*7.0cm*5.9cm), irregular, lobulated and space-occupying lesion in right parietooccipital lobe. Brain MRI after the operation; **(C, D)** No residual tumor in brain

The first patient, a 32-month-old boy, was first admitted on November 6, 2018 in Beijing Children’s Hospital with complaints of convulsion of left lower limb for a week. MRI examination of the brain revealed a giant (4.9cm*7.0cm*5.9cm), irregular, lobulated and space-occupying lesion in right parietooccipital lobe. The demarcation between lesion and right lateral ventricular wall and choroid was not clear. The lesion at local right tentorium cerebelli was enhanced intensely and the lesion at bilateral choroid plexus was also enhanced. (Fig. 1A, B) During all the time, The patient always had clear consciousness.

On November 13, 2018, the child received “tumor resection via right temporo-occipital craniotomy approach to the right third ventricle” in our Neurosurgery Department. He had a satisfactory postoperative course and postoperative gadolinium-contrast MRI revealed no residual tumor (Fig. 1C, D). The pathological results prompted splinter hemorrhage and necrosis in a portion of the tumor tissue. And the immunohistochemical results showed Vimentin (+), CK (AE1/AE3) (part +), ATRX (+), P53 (Scattered +), CEA (Scattered +), S-100 (Scattered +), and the maximum Ki67-labeling index as 10%. The final pathologic diagnosis was choroid plexus carcinoma (WHO Grade III).

On December 6, 2018, the 23th day after operation, the patient received eight cycles of chemotherapy through alternating intravenous application of CE (carboplatin and etoposide) and CV (cyclophosphamide and vincristine) programs, administered at 3 weeks intervals. During chemotherapy, liver/kidney function, aural comprehension ability and electrolyte were monitored regularly. All the results were within the normal range and no serious infection occurred. The children underwent MRI monitoring of the head and spinal cord every 3 cycles and at the end of chemotherapy, and the results were stable, no recurrence and metastasis were found. After the chemotherapy, the patient received whole-brain radiotherapy in another hospital in August 2019.

We also had follow-up with this patient. The MRI results of brain and spinal cord right after the radiotherapy and six months after the treatment showed no signs of tumor recurrence. By now, the child patient has normal growth and development without any special symptom.

### Case 2

**Fig. 2:**
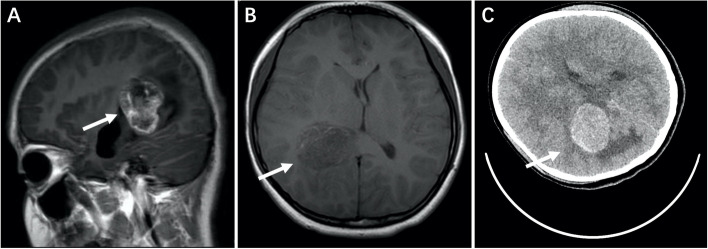
Brain MRI **(A, B)** and CT **(C)** in the Emergency Department before the operation. High-density space-occupying lesions were identified in the right temporal parietal lobe and the size was approximately 4cm*5cm*3cm

On April 19, 2019, a 13-year-old girl, the sister of the proband of Case 1, was admitted into the Emergency Department of our hospital with complaints of intermittent headache and dizziness for two weeks, accompanied by confusion and intermittent convulsions for 12 hours. Vomiting occurred after admission and soon she fell into a coma. Physical examination showed that the patient was unconscious, delirious, with slow pupil response to light. Her bilateral knee tendon reflexes were hyperactive and bilateral Babinski reflexes were positive. Then CT and MRI examination was urgently performed. High-density space-occupying lesions were identified in the right temporal parietal lobe and the size was approximately 4cm*5cm*3cm. Low-density edema zone was detected around the mass and the midline moved left under pressure. (Fig. 2)

Two hours after admission, the condition got worse for this child. Unequal pupils appeared and the right pupillary reaction to light was absent. She received “total resection of lateral ventricular tumors with craniotomy and corticostomy in the right triangle” immediately. The Pathology results were “high-grade glioma (right lateral ventricle)”. Histological morphology and immunophenotypic characteristics of the patients were highly consistent with symptoms of glioblastoma. Ependymal epithelial differentiation was accompanied locally (WHO Grade IV). The Immunohistochemistry showed following results: S-100(+), Vimentin(+), INI-1(+), GFAP(+), P53(-), D2-40(+), Syn(scattered, weak +), CK (AE1/AE3)(-), EMA(little, Nuclear side+), ATRX(-), H3K27M(-), IDH1(-), LIN28(-), NeuN(-), CD34(-), BRAF(-), SOX10(-), Olig2(+)RELA fusion gene (-)and the Ki-67 index as 40%+.

The child patient recovered well after operation. One month later, focal irradiation was initiated for her treatment in another hospital. The dose of external radiotherapy for whole brain was 36 Gy, administered by 20 times for a course of 4 weeks. And she did not receive chemotherapy with other drugs during this time. In July 2019, 1 month after radiotherapy, the patient was admitted to our hospital again for chemotherapy. Review of cranial MRI showed postoperative changes in the right parietal lobe, multiple abnormal signal shadows with bilateral temporal lobe, right insular cortex and straight back, and no signs of recurrence. Spinal cord MRI discovered no metastases. The child received a total of 9 courses of chemotherapy with Temozolomid. (oral administration for 5 days per cycle and each cycle lasting for 28 days). In this process, the dose of temozolomide was 150mg/m^2^ per day for the first cycle and was increased to 200mg/m^2^ per day for the second cycle. During the chemotherapy, no common adverse reactions such as nausea, vomiting and headache were observed in the child treated by temozolomide. In addition, no irreversible bone marrow depression and liver function injury was found throughout our monitoring.

**Fig. 3:**
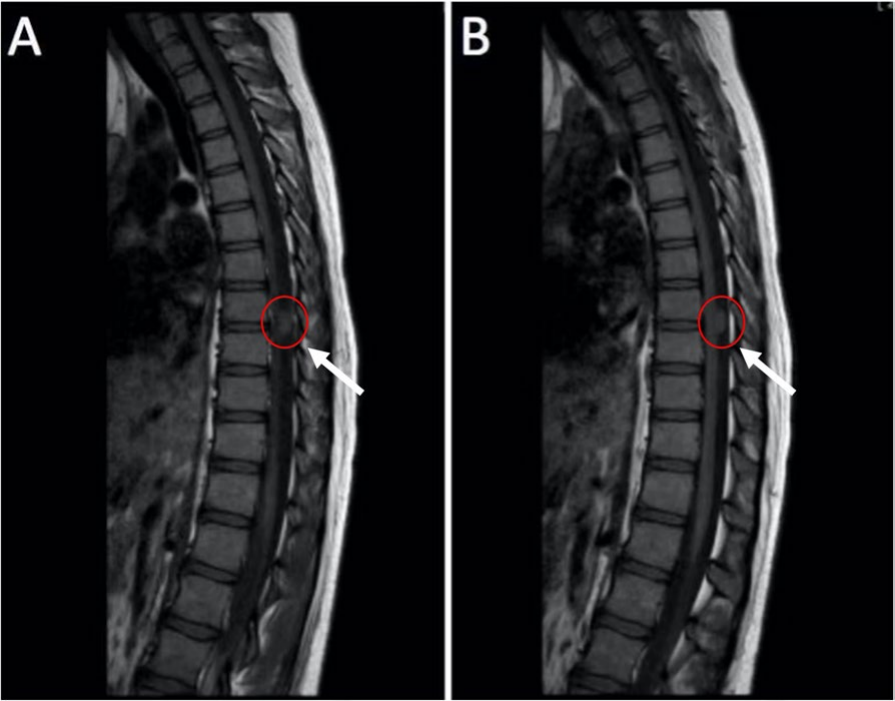
Small mass with abnormal signal at T8-9 on spinal MRI.

**Fig. 4:**
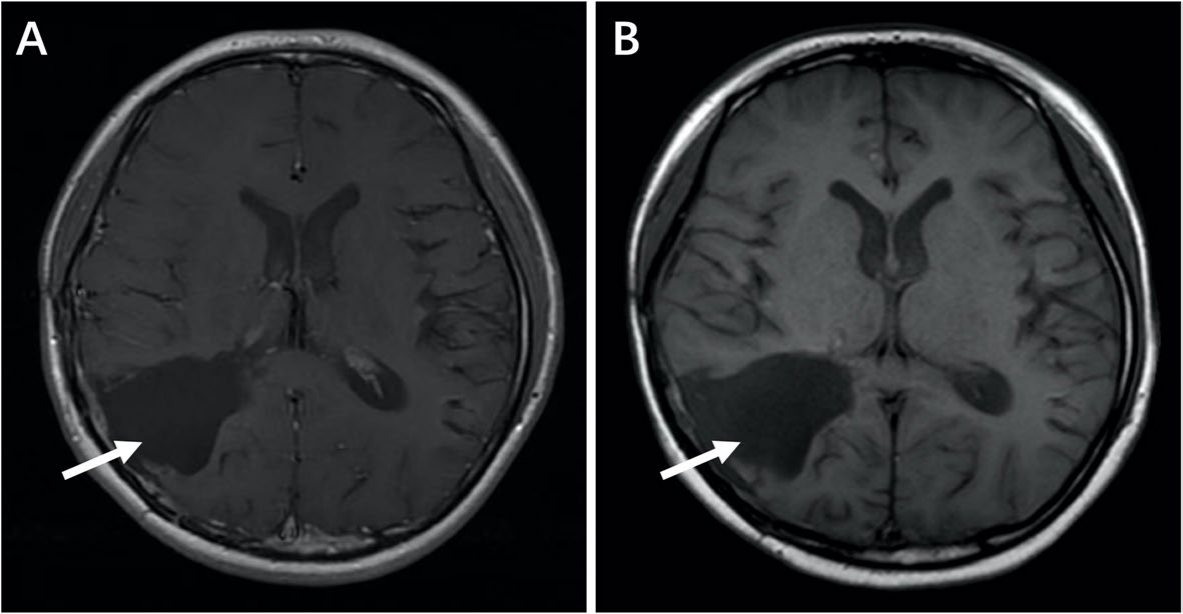
Brain MRI showed no significant change compared with previous **(A, B)**. **A** 2019.9; **B** 2020.04

During that time, cranial and spinal cord MRI were performed every three courses. No imaging signs suggested recurrence or metastasis of the tumor. Imaging examination of lungs and abdomen was also conducted regularly on the patient, and no metastasis or secondary tumor was found. But two months after the patient stopped temozolomide treatment, small mass with abnormal signal at T8-9 on spinal MRI (Fig. 3) was discovered by routine evaluation with no symptom accompanied. The brain was also checked and the MRI showed no significant change compared with previous (Fig. 4). About a month later, the patient received tumor resection of spinal cord in another hospital. The pathological diagnosis after operation was the same as before, suggesting spinal cord metastasis of glioblastoma. 25 days after the surgery, the patient continued treatment with Temozolomide by the original regimen (200mg/m^2^ per day), and also intrathecal injection of methotrexate. Unfortunately, the tumor progresses further and she is now undergoing palliative care in the local hospital.

## Genetics

Since two children in one family developed malignant tumors successively, and the brother suffered from the rare choroidal carcinoma, we decided to run a gene test for the whole family. The father, mother and another healthy brother are all in good physic status and they denied the history of tumor and family genetic disease.

**Fig. 5:**
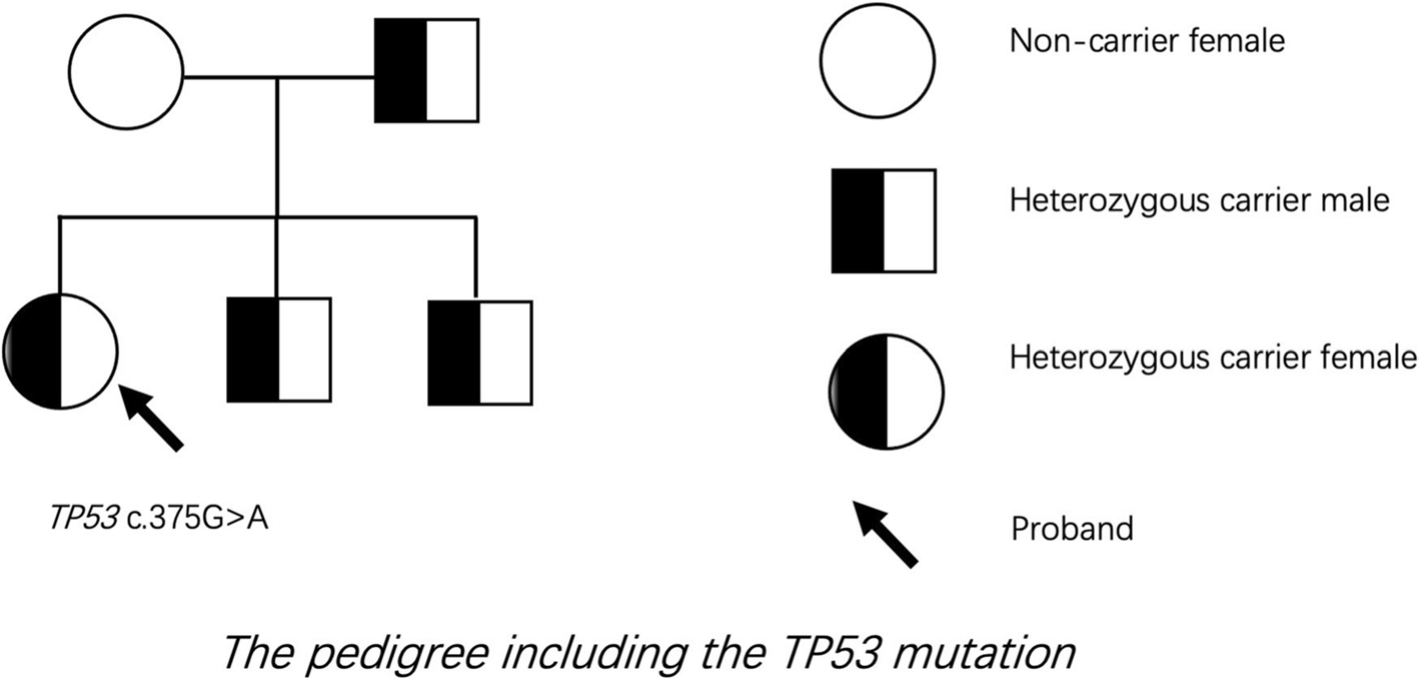
The pedigree including the TP53 mutation in this case

**Fig. 6:**
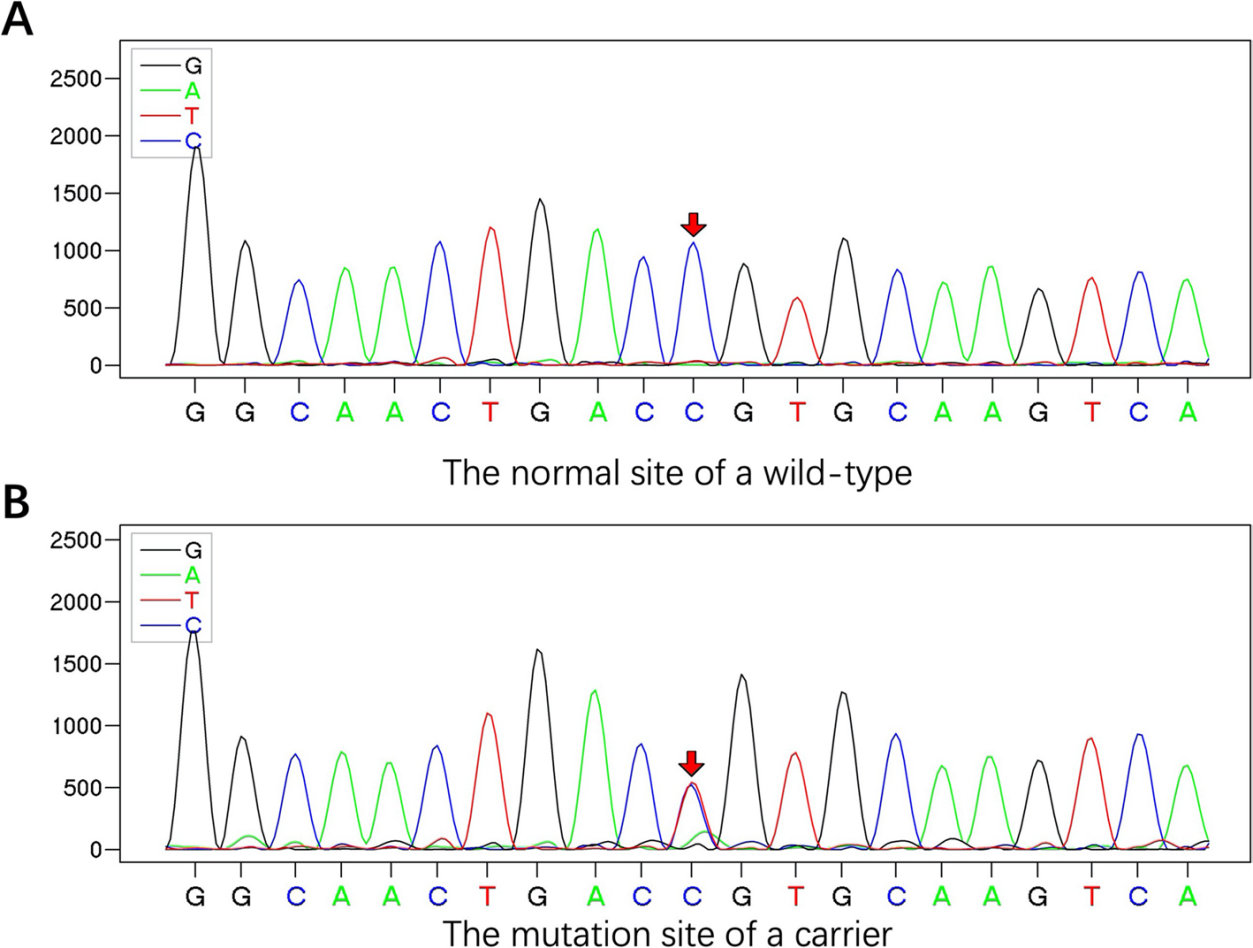
The DNA sequencing data around the mutation site from a wild-type control **(A)** and a carrier **(B)**

The genetic analysis confirmed that the TP53 gene mutation occurred in both children of these two cases. Their healthy brother also carries this mutation and this gene comes from the father of these children. The pedigree including the TP53 mutation is shown in Fig. 5, and the DNA sequencing data around the mutation site from the wild-type control and the carrier are shown in Fig. 6. The mutation site was c.375G > A, which is classified as a pathogenic variant according to the AGMG standard.

## Discussion and Conclusion

LFS is a typical hereditary tumor susceptibility syndrome with autosomal dominant inheritance, first discovered and reported by Frederick Pei Li and Joseph F. Fraumeni, Jr in 1969 [3]. LFS has been confirmed as associated with multiple tumors clinically and patients with LFS have high incidence of malignancies often with onset at the early age [4]. The two patient of our case both have the onset of childhood. According to the classic criteria [5], LFS is considered as present if the proband is diagnosed with sarcoma before 45 years old and a first/second-degree relative was diagnosed with any type of cancer or sarcoma before 45 years old. Recently, Chompret criteria has been suggested to clinically diagnose LFS [6]. In this criteria, tumors in the proband are not limited to sarcomas, also include brain tumor, breast cancer, and adrenocortical carcinoma(ACC). But they have younger age cutoff than the classical one. In case 1, the child was diagnosed as CPC at the age of two and his sister in case 2 was diagnosed as glioblastoma, which complied with the Chompret criteria.

**Fig. 7:**
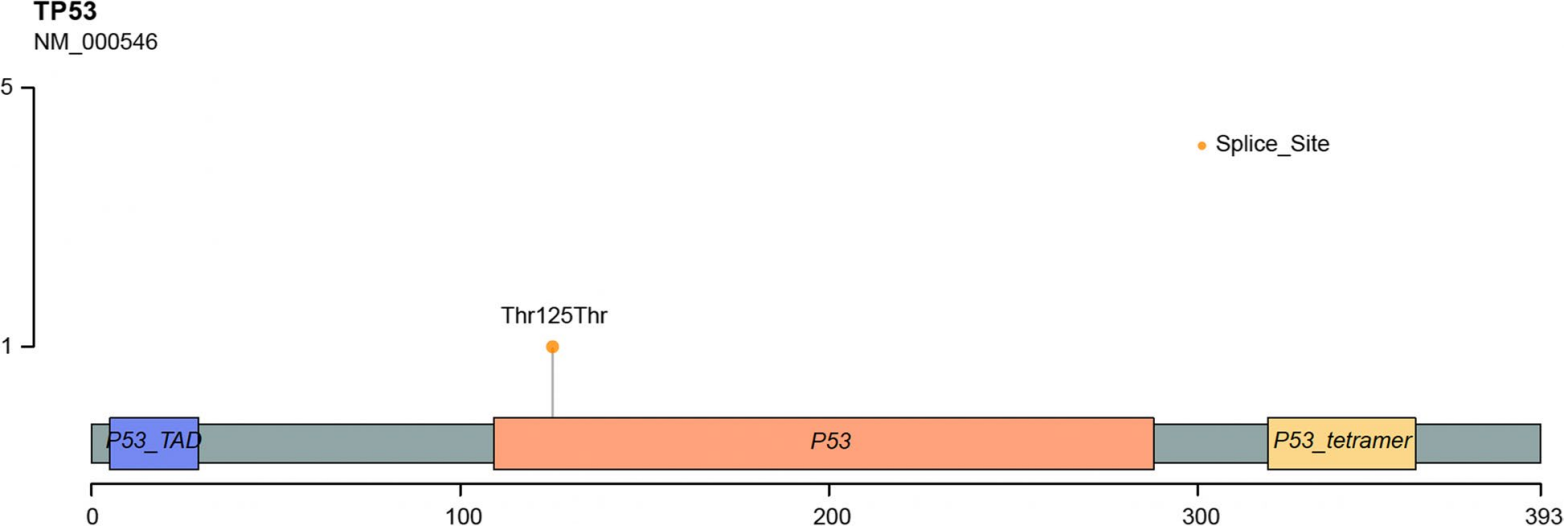
A diagram for TP53 protein with all known domains, and where the mutation is located

Germline mutations in the tumor suppressor gene TP53 on chromosome 17 are now widely recognized as the pathogenic gene of LFS [7]. This germline pathogenic mutations can be detected in 70%-80% of families with LFS [3]. The mutation site spectrum of TP53 is quite extensive and more than 1,800 different mutations have been reported by far. At present, six hotspot mutations have been found in 20% of patients with TP53 mutations, including p.R175H, p.G245S, p.R248Q, p.R248W, p.R273H and p.R282W [8, 9]. But in this case, the c.375G > A mutation, which the two patients carried, is non-hotspot mutation. The variant, located at the last base of exon 4. After the mutation, the amino acid change is p.Thr125Thr, which is actually a type of synonymous mutation and did not cause a change in the encoded amino acid. But the mutation will cause the donor site of the splice site to disappear, thereby affecting the splicing. We also drew a diagram for TP53 protein with all known domains, and indicate where this mutation is located (Fig. 7). This variant is included in the dbSNP database(rs55863639), and the frequency of this SNP in general population is 0.000007, but there is no frequency information in the gnomAD and ExAC databases, indicating that the frequency of the mutation population is extremely low. The Human Gene Mutation Database includes this variant as a pathogenic variant of LFS, in addition, the Clin var database includes this variant as a pathogenic variant for LFS based on the results of multiple submitters in clinical testing.. In summary, according to the AGMG standard, we classified the c.375G>A mutation of the TP53 gene as a pathogenic variant. We also used CADD and SIFT to predict pathogenesis and found that none of the different prediction methods had a score value, which may be related to the mutation being a synonymous mutation.

The most common cancers associated with LFS include osteosarcoma, ACC, CNS neoplasm and soft tissue sarcoma, with incidence rates of 30%, 27%, 26% and 23% respectively [10]. Both of our cases are brain tumors. The first one, CPC, is a rare pediatric brain neoplasm accounts for less than 1% of the total number of CNS tumors [11]. But with LFS, CPC are the classic brain tumor, glioma and medulloblastoma are also relevant to LFS [12]. In addition, A review study about Characteristics of LFS in Japan pointed out that Children with CPC of the brain and ACC were reported to have a high likelihood of carrying a TP53 germline variant, even in the absence of any family history [13]. Because in the early stage of the disease, the family members of the child denied the relevant family history of the tumor. For this reason We did not perform genetic testing for the first patient immediately, so we ignored their relevance and missed the opportunity to discover this LFS family earlier. Therefore, the report of this case also reminds clinicians to pay more attention to CPC, even when there is no family history of tumors, TP53 gene testing should be performed in time for children with CPC to be alert to the existence of LFS.

Thus, the diagnosis of LFS and the detection of TP53 mutation are very necessary. A large-sample clinical trial of Bougeard et al. included 1,730 patients with LFS in France, the results showed that the average age of first tumor was 24.9 years old, and 41% of the patients were clearly diagnosed as tumors before the age of 18 [10]. Another study had shown that the risk of primary cancer is 50% for women at the age of 31 and 50% for men at the age of 46 [14]. By analyzing the TP53 database of the international agency for research on cancer, the incidence rate of cancer in patients with TP53 mutation is estimated at 80% at 70 years old, while the risk of women is close to 100% due to the high incidence of breast cancer [15]. Therefore, we should be more sensitive to what kind of patients need genetic testing in clinical work, so that they and their families can be monitored for cancer earlier. The latest diagnostic criteria updated in 2009 by Chompret et al. defines conditions to select individuals who carry germline mutations in TP53 independent of family history [6, 16]. According to the current knowledge of TP53 gene, the following characteristics can be adopted as screening indicators for TP53 mutant genes in children: (1) various types of childhood tumors with at least one first/second-degree relative diagnosed with LFS-related tumors (premenopausal breast cancer, soft tissue sarcoma and ACC for example) at any age (2) At least one of all primary tumors in patients with multiple tumors in childhood belongs to the type of LFS-related tumors; (3) Once certain special types of tumors are diagnosed, TP53 gene detection should be performed for any of the following: breast cancer, ACC, CPC, papilloma, soft tissue sarcoma and osteosarcoma .

LFS patients with TP53 mutations have significantly higher rates of secondary tumors than those with normal TP53 [17]. Compared with adult patients, children may have a longer tumor development process because of their early-onset age. In order to manage related patients properly, once TP53 gene mutation is found positive with them, clinical monitoring should be carried out for early tumor detection and control of cancer and treatment-related morbidity and mortality [18]. Some scholars have found that the 5-year survival rate of LFS patients with TP53 mutation can be significantly improved through clinical monitoring [19]. Annual brain MRI and whole-body MRI were suggested for these children patients. Blood tests should be done regularly to check indicators like complete blood count, erythrocyte sedimentation rate, lactate dehydrogenase, testosterone, androstenedione and dehydroepiandrosterone sulfate. Abdominal and pelvic ultrasonography should be conducted. It is also important to have a complete physical examination for blood pressure, growth curve, Cushingoid appearance, male physical signs and comprehensive neurological assessment, with focus on rapid weight and height growth. Once LFS is diagnosed, family members should also be screened for related genetics and have a high probability of developing malignant neoplasm [20–23]. Early disease surveillance of TP53 mutation carried in family members of LFS patients since childhood can identify a wide range of early diagnosable diseases, including breast cancer, soft tissue sarcoma, brain tumor, osteosarcoma, ACC, bladder cancer, colorectal cancer, gastric cancer, nephroblastoma, liver cancer, lung cancer, melanoma and pancreatic cancer [24]. In our case, if we find that this is a LFS family at the first time, and carry out tumor surveillance on his family earlier, maybe we can find the sister’s glioblastoma sooner. The genes of the two children and their family members had already been tested since we realized this point. We followed up their father and another brother who carried the same mutant gene. Up to now, there were no signs of growing tumors. Furthermore, there are also recent literature reports that germ cell mosaicism as the cause of recurrence of LFS in siblings [25], so when the genetic test of the parent who was suspected of having a TP53 mutation, Even if the result is negative, we still cannot relax our vigilance against the siblings of the proband.

To develop mature treatment plans for LFS related patients, further exploration is also demanded to learn about the clinical characteristics of LFS patients with Tp53 mutation. The current study suggests that patients with TP53 gene mutation are very sensitive to radioactivity factors [12], Radiotherapy-induced cancer is more common in LFS patients [10]. Therefore, radiological examination and treatment should be avoided as much as possible in the process of diagnosis and treatment for such patients. But in our cases, both children received focal irradiation following gross total resection. In our opinion, prognostic benefits of postoperative radiotherapy must be weighed against the risk of long-term secondary cancer if the tumor is highly malignant. In addition, the results of Hendrickson et al. who studied 40 patients with LFS on radiotherapy and secondary malignant tumors were different from before, their data provide preliminary evidence to suggest RT should not be withheld in patients with LFS [26]. Recently, a strong link between TP53 mutations and hypermethylation at the promoter of the p53-associated microRNA miR-34A, which was found as a potential putative novel therapeutic target and a marker for clinical prognostication [27].

In conclusion, the germline mutation of the TP53 gene is the only known pathogenic gene of LFS. Timely identification and discovery of this mutation can effectively help clinicians to manage treatment and obtain the best therapeutic effect, especially for children. In such cases, aggressive preventive surveillance and treatment may bring optimistic outcome for patients and their family members who also carry this mutation.

## Abbreviations

LFS: Li-Fraumeni syndrome
CNS: Central nervous system
CPC: Choroid plexus carcinoma
CE: carboplatin and etoposide
CV: cyclophosphamide and vincristine
ACC: adrenocortical carcinoma
